# HEARING AID USE, COGNITIVE MEASURES, AND SPEECH-IN-NOISE PERFORMANCE: FINDINGS FROM THE ACHIEVE STUDY

**DOI:** 10.1093/geroni/igae098.0056

**Published:** 2024-12-31

**Authors:** Nicholas Reed, Kening Jiang, Alison Huang, Emmanuel Garcia Morales

**Affiliations:** Johns Hopkins University, Baltimore, Maryland, United States; Johns Hopkins University, Baltimore, Maryland, United States; Johns Hopkins Bloomberg School of Public Health, Baltimore, Maryland, United States; Cochlear Center for Hearing & Public Health, Baltimore, Maryland, United States

## Abstract

There is a paucity of information on how measures of cognition, brain structure, audition, and sociodemographic (e.g., education) affect changes in speech-in-noise performance, a priority for communication, with hearing aids over time. The ACHIEVE study is a randomized control trial of older adults with hearing loss enrolled to a hearing intervention or health education control over 3-years. Self-report demographic and health measures, social participation, hearing (pure-tone audiometry, Hearing Handicap Inventory for The Elderly[HHIE]), and a cognitive test battery (global and domain-specific [language; memory; executive function]) were collected at baseline. In addition, a subsample completed Magnetic Resonance Imaging (MRI) as part of an ancillary. Speech-in-noise performance (Quick Speech-in-Noise test; scores range 0-30 with higher being better) was measured at baseline without hearing aids and at 2-months and 3-years with hearing aids. Among 220 participants (median age=75.5; 50.9% Female) in the intervention arm, median unaided speech-in-noise scores were 19.0 (IQR=16.0-22.5) at baseline. At ~2-months and 3-years, aided scores were 21.3 (18.0-23.5) and 20.0 (16.0-22.5), respectively. Using linear mixed-effects models, age, education, martial status, social network, depressive symptoms, hypertension, hearing loss, HHIE, global, language domain and executive cognitive domains, temporal lobe volume, white matter hyperintensities, white matter integrity (fractional anisotropy, and mean diffusivity) were associated with baseline speech-in-noise performance. However, 2-month change was only associated with baseline hearing loss (B=0.28;95%CI=0.19-0.36), HHIE (B=0.12;95%CI=0.05-0.18), temporal lobe volume (B=0.93;95%CI=0.40-1.45), white matter hyperintensities (B=1.09;95%CI=0.43-1.76), fractional anisotrophy (B=-0.72;95%CI=-1.40--0.05), and mean diffusivity (B=0.70;95%CI=0.03-1.37) while 3-year change was only associated with baseline hearing (B=0.15;95%CI=0.04-0.26), HHIE (B=0.09;95%CI=0.01-0.19), and fractional anisotrophy (B=-1.14;95%CI=-2.08--0.20).

